# SNP rs3825214 in *TBX5* Is Associated with Lone Atrial Fibrillation in Chinese Han Population

**DOI:** 10.1371/journal.pone.0064966

**Published:** 2013-05-24

**Authors:** Xiaobiao Zang, Shulong Zhang, Yunlong Xia, Sisi Li, Fenfen Fu, Xiuchun Li, Fan Wang, Rongfeng Zhang, Xiaochen Tian, Lianjun Gao, Jiaying Zhang, Yanzong Yang, Xin Tu, Qing Wang

**Affiliations:** 1 First Affiliated Hospital of Dalian Medical University, Dalian, China; 2 Key Laboratory of Molecular Biophysics of the Ministry of Education, College of Life Science and Technology and Center for Human Genome Research, Cardio-X Institute, Huazhong University of Science and Technology, Wuhan, China; 3 Henan Provincial People's Hospital, Zhengzhou, China; Temple University, United States of America

## Abstract

**Background:**

A prolonged PR interval is a sign of increased risk of cardiac arrhythmia. Recent genome-wide association studies found that the single-nucleotide polymorphism (SNP) rs3825214 in T-box 5 (*TBX5*) was positively associated with PR interval, QRS duration, QT interval, and common arrhythmia disorders such as atrial fibrillation (AF) and advanced atrioventricular block. However, other independent replication studies are required to validate the result. This study assessed associations between rs3825214 and ECG parameters, AF, and ventricular tachycardia (VT) in a Chinese Han population.

**Methodology/Principal Findings:**

To assess the association between rs3825214 and AF and VT, we carried out case-control association studies with 692 AF patients (including 275 lone AF patients), 235 VT patients, and 856 controls. Genotyping was performed using a Rotor-Gene TM 6000 High Resolution Melt system. Statistical analyses of associations were adjusted for potential confounding factors. A moderate association was detected between rs3825214 and AF (*P*_adj_ = 0.036, OR = 0.79) and a highly significant association was detected between the G allele of rs3825214 and lone AF (*P*_adj_ = 0.001, OR = 0.65; genotypic *P* = 3.75×10^−4^ with a dominant model). We also found that rs3825214 showed a significant association with atrial-ventricular block (AVB; *P* = 0.028; *P*_adj_ = 0.035, OR = 0.494).

**Conclusions:**

Our results indicate that rs3825214 conferred a significant risk of lone AF in this Chinese Han population.

## Introduction

Atrial fibrillation (AF) is the most common supraventricular tachycardia encountered in clinical practice, and is associated with pronounced morbidity, mortality, and socio-economic burden [1]. Morphological and electrophysiological alterations that promote and maintain AF have been studied extensively, but the underlying mechanism is still not fully understood [2]. Some AF patients have a family history of the condition, and studies have suggested that genetic factors are involved in its development, especially in patients with lone AF [3], [4].

Ventricular tachycardia (VT), another important type of arrhythmia, is the most common cause of sudden cardiac death [5]. The previous studies found that many genetic factors of AF, such as genes encoding sodium or potassium ion channels, were shared by VT [6].

Recently, genome-wide association studies found that the single-nucleotide polymorphism (SNP) rs3825214, located in the T-box 5 (*TBX5*) gene, was positively associated with the electrocardiography (ECG) PR interval (combined *P* = 3.3×10^−12^), duration of the QRS complex (combined *P* = 3.0×10^−13^), and QT interval (combined *P* = 9.5×10^−8^) and may lead to cardiac arrhythmia such as AF and atrioventricular block (AVB) [7]. Functional studies also showed that *TBX5* is widely expressed in the atrial, atrioventricular node, and ventricular bundle branches, indicating that variations in *TBX5* may have an important role in the pathogenesis of AF and VT [8]–[13]. The present study explored the association between rs3825214 and ECG parameters PR, QRS, and QT intervals in a Han Chinese population, and the association between rs3825214 and two arrhythmia types, AF and VT.

## Methods

### Study subjects

Subjects were chosen from GeneID [14] and included 692 patients with AF, 235 patients with VT, and 856 controls. The Ethics Committee of First Affiliated Hospital of Dalian Medical University and Huazhong University of Science and Technology approved this study. All subjects provided informed written consent in accordance with the Declaration of Helsinki.

All study participants are of ethnic Han origin by self-report. AF patients over 70 years old were excluded to reduce confounding factors due to advanced age. Patients with other types of cardiac arrhythmias, hyperthyroidism, cardiomyopathies, and valvulopathies were excluded in the AF group. The evaluation of cardiac arrhythmias and other diseases were performed by at least two expert cardiologists.

The criteria for a diagnosis of AF were in accordance with established guidelines [15], and determined using a standard 12-lead ECG or Holter recordings, regardless of clinical symptoms. Lone AF was defined as AF occurring in patients younger than 60 years, without clinical or ECG evidence of cardiopulmonary disease, including hypertension. A diagnosis of VT rested on three or more consecutive ventricular premature beats regardless of ischemic or dilative cardiomyopathy, based on the analysis of a 12-lead ECG or Holter QRS morphology and rate [16]. Patients with primary cardiomyopathy, inherited cardiomyopathy were not included in this research.

The 856 control subjects were recruited from individuals who underwent annual physical exams with a normal ECG and without cardiac symptoms. We applied the criteria set by the American Diabetes Association to diagnose type 2 diabetes (T2DM) [17]. Evidence of coronary artery disease (CAD) was an angiograph showing more than 70% stenosis in at least one main vessel of the coronary artery. Patients with coronary artery bypass graft, percutaneous coronary intervention and/or myocardial infarction were classified as CAD subjects [18]. Stroke was defined in accordance with the World Health Organization's criteria of 1989 [19]. Hypertrophic cardiomyopathy was determined by excessive thickening of the left ventricular myocardium, occasionally also the right, without an identifiable cause such as arterial hypertension [20]. Hypertension was defined as clinical blood pressure of ≥140/90 mmHg or a history of medication.

### Genotyping

DNA was extracted from peripheral blood samples using the Wizard Genomic DNA Purification Kit (Promega). Genotypes with rs3825214 were determined via a polymerase chain reaction (PCR)-based high-resolution melting system with LC Green Plus fluorescent dye (RotorGene TM 6000, Corbett Life Science, Concorde, NSW, Australia) [21].

Primer pairs were designed based on the sequence of rs3825214 (forward, 5′-AGGGCTAACACCACCAGT-3′; reverse, 5′-AATGAGTCTGTGTTGAGGTTTA-3′). A total of 25 µL of PCR mixture was prepared, containing 1 µL of LC Green dye, 0.5 µL of 5 pmol of each primer, 25 ng of genomic DNA template, 2.5 µL of 10×PCR buffer, 0.5 µL of 5 mmol DNTPs, and one unit of DNA Taq polymerase. PCR was then performed at 95°C for 5 minutes; followed by 40 cycles of denaturation at 95°C for 10 seconds, annealing at 56°C for 10 seconds, and elongation at 72°C for 15 seconds; and finally 72°C for 7 minutes.

Two sequenced internal positive controls were set up for determining three genotypes (A/A, A/G and G/G). To validate the accuracy of genotyping, 50 samples (randomly selected) were used to directly sequence the region of rs3825214 using BigDye Terminator v3.1 Cycle Sequencing Kits on an ABI PRISM3100 Genetic Analyzer (Applied Biosystems, Foster City, CA, USA). The genotypes of sequenced samples were completely consistent with the high-resolution melting analysis.

### Statistical analysis

Student's *t*-test and Pearson's chi-squared (*χ* ^2^) test were used to analyze the differences in continuous traits (e.g., age) and categorical traits (e.g., gender, CAD, and hypertension) between the case and control groups, implemented with SPSS v17.0 software (IBM). The Hardy-Weinberg equilibrium was tested in the control group using PLINK v1.07 genetic analysis software (http://pngu.mgh.harvard.edu/~purcell/plink/download.shtml). A linear regression model was then used to test whether rs3825214 was associated with ECG parameters (PR, QRS, QT, and corrected QT [QT_c_] intervals). To test the association between rs3825214 and AF or VT, allelic analysis and genotypic analysis under three genetic models (dominant, recessive, and additive) were also applied. Odds ratios (ORs) and 95% confidence intervals (CI) were estimated using Pearson's *χ* ^2^ test. When the case-control samples were divided into several subgroups, Breslow-Day tests were performed to analyze the homogeneity between ORs from each subgroup (SPSS, 17.0). The Mantel-Haenszel test was performed to adjust for confounders. Multivariate logistic analysis was used to adjust for covariates such as age, gender, hypertension, CAD, stroke, and T2DM. To correct the data for multiple testing, the permutation test and Bonferroni correction were applied. Empirical *P*-values were determined using the PLINK v1.05 program with 100,000 Monte-Carlo permutations. Corrected *P*-values (*P*_cor_) were obtained using Bonferroni's correction according to the formula: *P*_cor_ = 1−(1−*P*_obs_)*^n^*, where *P*_obs_ is the observed *P*-value, and *n* is the number of comparisons. Statistical power analysis was conducted with the program PS (Power and Sample Size Calculations, version 3.0.43) [22]. A *P*-value<0.05 was considered statistically significant.

## Results

### Characteristics of subjects in the study

We studied 692 AF patients, 235 VT patients, and 856 controls (Table 1). The average age was 57±11 years for AF, 57±21 years for VT, and 56±15 years for controls. There are no significant differences in age or gender among these groups. Twenty-three VT patients (9.8%) also had AF (the rate of hypertension and T2DM are shown in Table 1). The hypertensive patients in the AF and VT groups numbered 266 and 89 respectively, and 175 and 67 had CAD. In the AF group, 45 patients had T2DM, and 25 had T2DM in the VT group. The number of patients with both AF and stroke was 87. Twenty-three patients had both AF and VT.

**Table 1:** Clinical characteristics of patients in GeneID population.

	AF group			VT group		
	AF	Control	*P*	VT	Control	*P*
	(n = 692)	(n = 856)		(n = 235)	(n = 856)	
Gender (male/female)	424/259	543/310	0.524	135/91	543/310	0.278
Age (mean ± SD, y)	57±11	56±15	0.724	57±21	56±15	0.583
Lone AF (%)	275 (39.7%)	N/A	-	N/A	N/A	—
Other AF (%)	417 (60.3%)	N/A	-	23 (9.8%)	N/A	—
Hypertension (%)	266 (38.4%)	60(7.0%)	<0.01	89 (37.9%)	60(7.0%)	<0.01
CAD (%)	175 (25.3%)	N/A	-	67 (28.5%)	N/A	—
T2DM (%)	45 (6.5%)	8(0.9%)	<0.01	25 (10.6%)	8(0.9%)	<0.01
Stroke history (%)	87 (12.6%)	N/A	-	26 (11.1%)	N/A	—

AF, atrial fibrillation; CAD, coronary artery disease; T2DM, type 2 diabetes; N/A, data not available.

### Association of rs3825214 with four ECG parameters

In this study, we performed an association analysis between the presence of rs3825214 and four ECG parameters (the PR, QRS, QT, and QT_c_ intervals), using the program PLINK for the AF, VT, and control groups (Table 2). In contrast to the results of other genome-wide association studies [7], we found no significant association between rs3825214 and the PR, QRS, or QT intervals, but the PR interval did show a tendency toward association (*P* = 0.057). Of note, we observed an association between rs3825214 and QT_c_ (*P* = 0.047).

**Table 2:** Association of SNP rs3825214 with ECG measures.

ECG measures (ms)	BETA	SE	*P*
PR interval	−6.353	3.324	0.057
QRS duration	−18.88	31.02	0.546
QT interval	−2.754	4.345	0.527
QT_c_ interval	−19.76	9.693	0.047

BETA, regression coefficient; SE, standard error.

### Allelic association of G allele of rs3825214 with AF and AVB

Comparisons of the minor allele frequencies of rs3825214 among the groups are summarized in Table 3. There was no deviation from the Hardy-Weinberg equilibrium for rs3825214 in the control group (*P* = 0.8361). Neither of the genders was associated with minor allele G of rs3825214. There was a significant association between the G allele and AF (*P* = 0.036; *P*_adj_ = 0.025, OR = 0.790) and between the G allele and AVB (*P* = 0.028; *P*_adj_ = 0.035, OR = 0.494) even after adjusting for age, gender, hypertension, CAD, stroke, and T2DM by logistic regression. A significant association between minor allele G of rs3825214 and lone AF was found (*P* = 0.002; *P*_adj_ = 0.001, OR = 0.652), which also survived the Bonferroni correction, while analysis of the other AF(AF outside of lone AF) showed no significant association (*P* = 0.546). Stratified analysis using the Breslow-Day test showed heterogeneity as measured by OR in the two subgroups (*P* = 0.049). Hence, among AF cases, the preponderance of associations between minor allele frequencies and AF was due to lone AF. When considering all patients with VT, there was no significant association between this condition and other cardiovascular diseases.

**Table 3:** Allelic analysis of SNP rs3825214 association with AF and VT.

Cohort (cases/controls)	Frequency of G allele (cases/controls)	*P*-obs	*P*-adj	OR (95% CI)
**AF group**				
Male (424/543)	0.419/0.455	0.117	0.091	0.81 (0.63–1.04)
Female (259/310)	0.407/0.450	0.148	0.143	0.76 (0.53–1.10)
Total AF (692/856)	0.414/0.452	0.036	0.025	0.79 (0.64–0.97)
Lone AF (275/856)	0.376/0.452	0.002	0.001	0.65 (0.50–0.84)
Other AF (417/856)	0.439/0.452	0.546	0.631	1.08 (0.80–1.45)
CAD (175/856)	0.446/0.452	0.927	0.361	0.80 (0.49–1.30)
Hypertension (266/796)	0.417/0.467	0.364	0.001	0.59 (0.43–0.81)
T2DM (45/848)	0.478/0.456	0.683	0.134	2.62 (0.74–9.27)
Stroke (87/856)	0.489/0.452	0.351	0.558	1.25 (0.60–2.62)
**VT group**				
Male (144/543)	0.448/0.455	0.833	0.653	1.10 (0.73–1.66)
Female (91/310)	0.390/0.450	0.152	0.774	1.08 (0.64–1.83)
Total VT (235/856)	0.426/0.452	0.315	0.712	1.06 (0.77–1.47)
AVB (24/856)	0.292/0.452	0.028	0.035	0.49 (0.26–0.95)
BBB (29/856)	0.431/0.452	0.758	0.371	1.55 (0.59–4.03)
Bradycardia (27/856)	0.463/0.452	0.868	0.684	1.25 (0.42–3.71)
ER (12/856)	0.500/0.452	0.636	0.661	1.34 (0.37–4.89)
HCM (26/856)	0.365/0.452	0.219	0.674	1.20 (0.51–2.82)
MVA (61/856)	0.385/0.452	0.155	0.469	1.25 (0.69–2.27)

BBB: bundle branch block; ER: early repolarization; HCM: hypertrophic cardiomyopathy; MVA: malignant ventricular arrhythmia.

### Genotypic association of rs3825214 with AF

**Figure 1:**
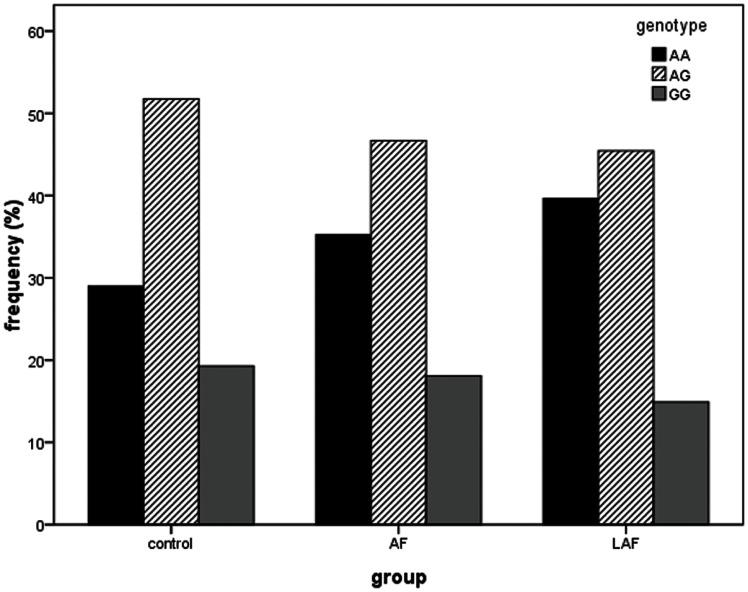
Genotype distributions of rs3825214 in patients with AF and lone AF.

The genotype distributions of rs3825214 among the groups are shown in Table 4. In both the all AF and lone AF groups, the distributions of rs3825214 were significantly different compared with the control group (*P* = 0.029 and 0.003, respectively). No significant association was observed between genotypes carrying rs3825214 and ECG parameters. A bar chart of genotype distributions shows that the genotype GG made up the smallest proportion among the three genotypes (Fig. 1). Indeed, assuming a dominant genetic model, GG was strongly associated with AF, and especially lone AF (Table 5). The Bonferroni correction was adjusted for multiple testing. Empirical significance values were observed under dominant and additive models in the total AF and lone AF groups (OR was 0.73 after adjusting for gender, age, T2DM, hypertension, stroke, and CAD).

**Table 4:** The genotype distributions of rs3825214 in different groups.

	AA	AG	GG	*P*
Gender (M/F)	233/121	356/207	132/74	0.728
Age	49.36±16.8	49.47±16.0	49.11±15.5	0.964
Lone AF	109	125	41	0.003
Total AF	244	323	125	0.029
VT	81	108	46	0.214
HR (bpm)	84.01±24.4	79.30±29.9	80.16±32.4	0.565
PR interval (ms)	174.84±48.3	160.74±32.5	161.95±31.2	0.113
QRS duration (ms)	107.67±25.8	137.00±94.6	120.67±61.5	0.492
QT interval (ms)	381.99±50.4	383.81±46.1	375.71±49.2	0.607
QT_c_ interval (ms)	442.29±66.8	434.60±41.2	447.22±30.7	0.754

**Table 5:** Genotypic analysis of SNP rs3825214 with AF under three genetic models.

	*P-obs*	*P-cor* a	*P-adj* b	OR (95% CI)	*P-emp* c
**Total AF**					
Dominant	0.008	0.016	0.008	0.82 (0.70–0.95)	0.011
Recessive	0.543	0.791	0.406	0.86 (0.59–1.24)	0.529
Additive	0.029	/	0.069	0.77 (0.58–1.02)	0.028
**Lone AF**					
Dominant	9.32×10^−4^	0.004	3.75×10^−4^	0.73 (0.61–0.87)	0.001
Recessive	0.103	0.353	0.123	0.83 (0.65–1.05)	0.173
Additive	0.003	0.006	0.034	0.69 (0.49–0.97)	0.003
**Other AF**					
Dominant	0.214	0.618	0.884	1.02 (0.81–1.28)	0.245
Recessive	0.714	0.993	0.506	1.09 (0.85–1.40)	0.583
Additive	0.333	0.666	0.688	0.92 (0.61–1.39)	0.314
**VT**					
Dominant	0.104	0.208	0.943	0.99 (0.78–1.27)	0.099
Recessive	0.918	1.836	0.468	1.11 (0.84–1.45)	0.857
Additive	0.214	/	0.520	0.86 (0.55–1.35)	0.197

a *P*-cor values were obtained using Bonferroni's correction.

b *P*-adj was obtained using multivariable logistic regression analysis by including covariates of gender, age, T2DM, hypertension, and CAD.

c *P-*emp empirical *P* values obtained by performing 100,000 permutations.

## Discussion

In the present study, we assessed the association of rs3825214 with four ECG parameters (PR, QRS, QT, and QT_c_ intervals) and two types of arrhythmias (AF and VT) in a Chinese Han population. Consistent with previous studies [7], we found that rs3825214 was associated with AF and AVB. However, we did not find any association between rs3825214 and PR interval, QRS duration, or QT interval. Notably, for the first time to our knowledge, we found that rs3825214 was associated with QT_c_, and conferred risk of lone AF in Han Chinese.

In an earlier study, the investigators were able to derive a predictor of postoperative AF or atrial flutter by measuring total P wave duration obtained from the simultaneous recording of the three standard limb leads [23]. Recently, some longitudinal studies also showed that a prolonged PR interval was linked to increased incidence of AF [24]–[27]. The PR interval could alter the duration of atrial action potential and atrioventricular conduction in AF patients. Thus, the PR interval was considered an endophenotype for AF [28]. Nevertheless, as an established risk factor for AF, the PR interval length was not consistently influenced by the presence of SNPs [29]. In our study, rs3825214 was significantly associated with AVB, including advanced AV block, mild AV block (*P* = 0.028, *P*_adj_ = 0.035, OR = 0.494), and AF, but not with the PR interval (*P* = 0.113).

Lone AF patients are those with no detectable cardiovascular disease, about 30% of AF cases [30]. Genetic factors are assumed to be important to the development of lone AF [31]. In the present study, when we divided the total AF group into the subgroups lone AF and other AF, the association between rs3825214 and lone AF was much more significant than for the AF group as a whole (OR = 0.65, *P* = 0.002). The association for GG frequencies of rs3825214 with lone AF is even more significant with the assumption of a dominant genetic model (*P* = 3.75×10^−4^) after adjusting for possible risk factors, including gender, age, T2DM, hypertension, and CAD.

The longer QT interval, which represents myocardial repolarization, is associated with a lowered ventricular arrhythmia threshold and is a risk factor for sudden cardiac death and drug-induced arrhythmias. In this study, the results also showed that rs3825214 was associated with QT_c_ (*P* = 0.047, BETA = −19.76) but not between rs3825214 and VT.

As a member of the T-box transcription factor family, *TBX5* is important to heart and limb development. Mutation of *TBX5* is the main cause of Holt-Oram syndrome, a hereditary disease characterized by forelimb and cardiac congenital defects such as atrial and ventricular septal defect [8]. Moreover, *TBX5* is required for the patterning and maturation of the murine cardiac conduction system and is widely expressed in the atrioventricular node and ventricular bundle branches in mice [9], [10]. *TBX5* may affect the development of paroxysmal AF, based on the research of a large atypical Holt-Oram syndrome family [11]. Combined with the results of population genetics studies and functional experiments, this suggests that variations in *TBX5* may affect the cardiac conduction system and is involved in the pathogenesis of AF.

Our study has several limitations. (1) Although an association between rs3825214 and lone AF is demonstrated for the first time, the power to detect an association was only 60%, with an OR of 0.73 with a type I error probability of 0.05. Further studies with larger sample sizes are needed to further assess the association. (2) The diagnosis of lone AF needs to exclude more carefully other diseases that could initially present as lone AF. Though we set strict inclusion criteria for lone AF patients when using the GeneID database, many of the recorded lone AF cases are linked to other factors that are not well known [32].

In conclusion, the present study was the first to provide evidence that rs3825214 in *TBX5* is associated with lone AF and prolonged QT_c_ in a Chinese Han population.

## References

[1] Go AS, Hylek EM, Phillips KA, Chang Y, Henault LE, et al (2001) Prevalence of diagnosed atrial fibrillation in adults: national implications for rhythm management and stroke prevention: the AnTicoagulation and Risk Factors in Atrial Fibrillation (ATRIA) Study. JAMA 285: 2370–2375. 11343485 10.1001/jama.285.18.2370

[2] Schotten U, Verheule S, Kirchhof P, Goette A (2011) Pathophysiological mechanisms of atrial fibrillation: a translational appraisal. Physiol Rev 91: 265–325. 21248168 10.1152/physrev.00031.2009

[3] Kopecky SL, Gersh BJ, McGoon MD, Whisnant JP, Holmes DR Jr, et al (1987) The natural history of lone atrial fibrillation:A population-based study over three decades. N Engl J Med 317: 669–674. 3627174 10.1056/NEJM198709103171104

[4] Marcus GM, Smith LM, Vittinghoff E, Tseng ZH, Badhwar N, et al (2008) A first-degree family history in lone atrial fibrillation patients. Heart Rhythm 5: 826–830. 18468961 10.1016/j.hrthm.2008.02.016 PMC2474569

[5] Chopra N, Knollmann BC (2011) Genetics of Sudden Cardiac Death Syndromes. Curr Opin Cardiol 26: 196–203. 21430528 10.1097/HCO.0b013e3283459893 PMC3145336

[6] Prystowsky EN, Padanilam BJ, Joshi S, Fogel RI (2012) Ventricular Arrhythmias in the Absence of Structural Heart Disease. J Am Coll Cardiol 59: 1733–1744. 22575310 10.1016/j.jacc.2012.01.036

[7] Holm H, Gudbjartsson DF, Arnar DO, Thorleifsson G, Thorgeirsson G, et al (2010) Several common variants modulate heart rate, PR interval and QRS duration. Nat Genet 42: 117–122. 20062063 10.1038/ng.511

[8] Li QY, Newbury-Ecob RA, Terrett JA, Wilson DI, Curtis AR, et al (1997) Holt-Oram syndrome is caused by mutations in TBX5, a member of the Brachyury (T) gene family. Nat Genet 15: 21–29. 8988164 10.1038/ng0197-21

[9] Moskowitz IP, Pizard A, Patel VV, Bruneau BG, Kim JB, et al (2004) The T-Box transcription factor Tbx5 is required for the patterning and maturation of the murine cardiac conduction system. Development 131: 4107–4116. 15289437 10.1242/dev.01265

[10] Ghosh TK, Song FF, Packham EA, Buxton S, Robinson TE, et al (2009) Physical interaction between TBX5 and MEF2C is required for early heart development. Mol Cell Biol 29: 2205–2218. 19204083 10.1128/MCB.01923-08 PMC2663302

[11] Postma AV, van de Meerakker JB, Mathijssen IB, Barnett P, Christoffels VM, et al (2008) A gain-of-function TBX5 mutation is associated with atypical Holt-Oram syndrome and paroxysmal atrial fibrillation. Circ Res 102: 1433–1442. 18451335 10.1161/CIRCRESAHA.107.168294

[12] Hodgson-Zingman DM, Karst ML, Zingman LV, Heublein DM, Darbar D, et al (2008) Atrial natriuretic peptide frameshift mutation in familial atrial fibrillation. N Engl J Med 359: 158–165. 18614783 10.1056/NEJMoa0706300 PMC2518320

[13] Gollob MH, Jones DL, Krahn AD, Danis L, Gong XQ, et al (2006) Somatic mutations in the connexin 40 gene (GJA5) in atrial fibrillation. N Engl J Med 354: 2677–2688. 16790700 10.1056/NEJMoa052800

[14] Wang F, Xu CQ, He Q, Cai JP, Li XC, et al (2011) Genome-wide association identifies a susceptibility locus for coronary artery disease in the Chinese Han population. Nat Genet 43: 345–349. 21378986 10.1038/ng.783

[15] Fuster V, Rydén LE, Cannom DS, Crijns HJ, Curtis AB, et al (2006) ACC/AHA/ESC 2006 guidelines for the management of patients with atrial fibrillation: full text: a report of the American College of Cardiology/American Heart Association Task Force on practice guidelines and the European Society of Cardiology Committee for Practice Guidelines (Writing Committee to Revise the 2001 guidelines for the management of patients with atrial fibrillation) developed in collaboration with the European Heart Rhythm Association and the Heart Rhythm Society. Europace 8: 651–745. 16987906 10.1093/europace/eul097

[16] Aliot EM, Stevenson WG, Almendral-Garrote JM, Bogun F, Calkins CH, et al (2009) EHRA/HRS Expert Consensus on Catheter Ablation of Ventricular Arrhythmias: developed in a partnership with the European Heart Rhythm Association (EHRA), a Registered Branch of the European Society of Cardiology (ESC), and the Heart Rhythm Society (HRS); in collaboration with the American College of Cardiology (ACC) and the American Heart Association (AHA). Europace 11: 771–817. 19443434 10.1093/europace/eup098

[17] Cheng X, Shi L, Nie S, Wang F, Li X, et al (2011) The same chromosome 9p21.3 locus is associated with type 2 diabetes and coronary artery disease in a Chinese Han population. Diabetes 60: 680–684. 21270277 10.2337/db10-0185 PMC3028370

[18] Xu C, Wang F, Wang B, Li X, Li C, et al (2010) Minor allele C of chromosome 1p32 single nucleotide polymorphism rs11206510 confers risk of ischemic stroke in the Chinese Han population. Stroke 41: 1587–1592. 20576952 10.1161/STROKEAHA.110.583096

[19] Goldstein M, Barnett HJM, Orgogozo JM (1989) Recommendations on stroke prevention, diagnosis, and therapy. Report of the WHO Task Force on stroke and other cerebrovascular disorders. Stroke 20: 1407–1431. 2799873 10.1161/01.str.20.10.1407

[20] Elliott P, Andersson B, Arbustini E, Bilinska Z, Cecchi F, et al (2008) Classification of the cardiomyopathies: a position statement from the European Society of Cardiology Working Group on Myocardial and Pericardial Diseases. Eur Heart J 29: 270–276. 17916581 10.1093/eurheartj/ehm342

[21] Wittwer CT (2009) High-resolution DNA melting analysis: advancements and limitations. Hum Mutat 30: 857–859. 19479960 10.1002/humu.20951

[22] Dupont WD, Plummer WD Jr (1998) Power and Sample Size Calculations for studies Involving Linear Regression. Controlled Clinical Trials 19: 589–601. 9875838 10.1016/s0197-2456(98)00037-3

[23] Buxton AE, Josephson ME (1981) The role of P-wave duration as a predictor of postoperative atrial arrhythmias. Chest 80: 68–73. 6972856 10.1378/chest.80.1.68

[24] Cheng S, Keyes MJ, Larson MG, McCabe EL, Newton-Cheh C, et al (2009) Long-term outcomes in individuals with prolonged PR interval or first-degree atrioventricular block. JAMA 301: 2571–2577. 19549974 10.1001/jama.2009.888 PMC2765917

[25] Soliman EZ, Prineas RJ, Case LD, Zhang ZM, Goff DC Jr (2009) Ethnic distribution of ECG predictors of atrial fibrillation and its impact on understanding the ethnic distribution of ischemic stroke in the Atherosclerosis Risk in Communities (ARIC) study. Stroke 40: 1204–1211. 19213946 10.1161/STROKEAHA.108.534735 PMC2685189

[26] Schnabel RB, Sullivan LM, Levy D, Pencina MJ, Massaro JM, et al (2009) Development of a risk score for atrial fibrillation (the Framingham Heart Study): a community-based cohort study. Lancet 373: 739–745. 19249635 10.1016/S0140-6736(09)60443-8 PMC2764235

[27] Macfarlane PW, Murray H, Sattar N, Stott DJ, Ford I, et al (2011) The incidence and risk factors for new onset atrial fibrillation in the PROSPER study. Europace 13: 634–639. 21325345 10.1093/europace/eur016

[28] Goodloe AH, Herron KJ, Olson TM (2011) Uncovering an Intermediate Phenotype Associated With rs2200733 at 4q25 in Lone Atrial Fibrillation. Am J Cardiol 107: 1802–1805. 21481830 10.1016/j.amjcard.2011.02.326

[29] Pfeufer A, van Noord C, Marciante KD, Arking DE, Larson MG, et al (2010) Genome-wide association study of PR interval. Nat Genet 42: 153–159. 20062060 10.1038/ng.517 PMC2850197

[30] Kozlowski D, Budrejko S, Lip GY, Rysz J, Mikhailidis DP, et al (2010) Lone atrial fibrillation: what do we know? Heart 96: 498–503. 19713203 10.1136/hrt.2009.176321

[31] Marcus GM, Smith LM, Vittinghoff E, Tseng ZH, Badhwar N, et al (2008) A first-degree family history in lone atrial fibrillation patients. Heart Rhythm 5: 826–830. 18468961 10.1016/j.hrthm.2008.02.016 PMC2474569

[32] Frost L (2007) Lone atrial fibrillation: good, bad, or ugly? Circulation 115: 3040–3041. 17576879 10.1161/CIRCULATIONAHA.107.709287

